# Prediction of Pesticide Interactions with Proteins Involved in Human Reproduction by Using a Virtual Screening Approach: A Case Study of Famoxadone Binding CRBP-III and Izumo

**DOI:** 10.3390/ijms25115790

**Published:** 2024-05-26

**Authors:** Fabiana Tortora, Valentina Guerrera, Gennaro Lettieri, Ferdinando Febbraio, Marina Piscopo

**Affiliations:** 1 Institute of Genetics and Biophysics “Adriano Buzzati Traverso”, National Research Council (CNR), Via P. Castellino 111, 80131 Naples, Italy; fabiana.tortora@igb.cnr.it; 2 Institute of Biochemistry and Cell Biology, National Research Council (CNR), Via P. Castellino 111, 80131 Naples, Italy; 3 Department of Biology, University of Naples Federico II, Via Cinthia, 21, 80126 Naples, Italymarina.piscopo@unina.it (M.P.)

**Keywords:** human reproduction, molecular docking, virtual screening, pesticides, cellular retinol binding protein-III, IZUMO sperm–egg fusion protein, famoxadone

## Abstract

In recent years, the awareness that pesticides can have other effects apart from generic toxicity is growing. In particular, several pieces of evidence highlight their influence on human fertility. In this study, we investigated, by a virtual screening approach, the binding between pesticides and proteins present in human gametes or associated with reproduction, in order to identify new interactions that could affect human fertility. To this aim, we prepared ligand (pesticides) and receptor (proteins) 3D structure datasets from online structural databases (such as PubChem and RCSB), and performed a virtual screening analysis using Autodock Vina. In the comparison of the predicted interactions, we found that famoxadone was predicted to bind Cellular Retinol Binding Protein-III in the retinol-binding site with a better minimum energy value of −10.4 Kcal/mol and an RMSD of 3.77 with respect to retinol (−7.1 Kcal/mol). In addition to a similar network of interactions, famoxadone binding is more stabilized by additional hydrophobic patches including L20, V29, A33, F57, L117, and L118 amino acid residues and hydrogen bonds with Y19 and K40. These results support a possible competitive effect of famoxadone on retinol binding with impacts on the ability of developing the cardiac tissue, in accordance with the literature data on zebrafish embryos. Moreover, famoxadone binds, with a minimum energy value between −8.3 and −8.0 Kcal/mol, to the IZUMO Sperm–Egg Fusion Protein, interacting with a network of polar and hydrophobic amino acid residues in the cavity between the 4HB and Ig-like domains. This binding is more stabilized by a predicted hydrogen bond with the N185 residue of the protein. A hindrance in this position can probably affect the conformational change for JUNO binding, avoiding the gamete membrane fusion to form the zygote. This work opens new interesting perspectives of study on the effects of pesticides on fertility, extending the knowledge to other typologies of interaction which can affect different steps of the reproductive process.

## 1. Introduction

Human reproduction is a complex process constituted by many interconnected biological pathways, involving numerous molecular processes as well as interactions between gametic cells (sperm and egg cells), and it represents one of the most important steps of human life [1,2]. In mammals, fertilization involves a series of sequential steps, starting with sperm/oocyte membrane recognition and fusion [1]. When this recognition between gametes does not happen or happens incorrectly, infertility occurs [3]. Worldwide, approximately 50 million couples of reproductive age are unable to conceive, and the prevalence of infertility among couples is estimated to be between 10% and 20% [4]. Some endogenous factors, such as physical and structural problems affecting egg cells [5,6] or provoking hormone alterations [7], are known to determine serious impairments to female fertility, but, in about 15–20% of women, there is no evidence to explain infertility. Similarly, male factor infertility has been attributed to a variety of clinical, hormonal, and genetic causes; however, nearly 30% of men have impaired sperm parameters with no identifiable cause, even in the most comprehensive real-world diagnostic work-up currently available, defining idiopathic male infertility. Recent evidence suggests that idiopathic male infertility are linked to an array of unidentified pathologies that affect the testicular microenvironment and sperm properties, such as the exposure to environmental toxins and reactive oxygen species (ROS), causing DNA damage and genetic/epigenetic changes that reduce overall sperm quality and fertility potential [8]. In recent decades, industrialization has been a key factor in the spread of pollution, going on to impact many aspects of human health, including human fertility [9]. As evidence, male infertility has risen from 8% in the 1960s to 30% today in many industrialized countries [10,11], posing a relationship with the increase in industrial pollution [9]. Indeed, several studies report how industrialization in large cities is implicated in the degradation of semen quality; in particular, the air pollution (PM2.5, PM10, PAHs, O_3_, and NOx) by industries, is associated with an increase in abnormal sperm, reduced motility, DNA fragmentation, and hormone disruption [12]. The exposure to pollutants, such as dioxins, pesticides, heavy metals, microplastics, cigarette smoke, and agricultural additives, i.e., phthalates and bisphenol, is constant, and, more frequently than not, the mechanisms underlying their toxicity on the reproductive system are not entirely clear [13,14,15]. Some of these substances are already known to cause reproductive harm, because they are similar to natural molecules which regulate testicular development, sexual differentiation, sperm production, and testosterone in males. Especially in women, cigarette smoke increases the production of ROS and interferes with the levels of hormones involved in ovulation [12]. These molecules could act on the endocrine system, inducing the development of various alterations related to the reproductive system, causing a failure of fertilization [16,17]. Despite the large numbers of in vivo and in vitro studies on the toxic effects of several chemicals, it remains difficult still to identify the molecular processes associated with negative effects on human reproduction. In this regard, we have demonstrated the involvement of environmental pressure in the oxidative DNA damage of the sperm nuclear basic proteins (SNBPs) in human spermatozoa of healthy young men living in polluted areas [18] and we took a first look at the possible transgenerational effects of pollutants in the spermatozoa of fathers and sons in areas of high environmental impact [19]. In addition, new industrially synthesized molecules could underlie new causes of idiopathic infertility [20,21], so targeted studies are required so that we can understand what the consequences of these pollutants might be, once introduced in the environment. In this scenario, where new chemicals are constantly introduced by man-made sources of pollution and insufficient, time-consuming, and expensive experimental studies have been performed on a limited number of the ones already present in the environment, new approach methodologies (NAMs) are required in order to speed up or better address the study on the effects of these chemicals on human health, in particular, on fertility. NAMs are any technology, method, approach, or combination that can be used to provide information about chemical hazards and potential human exposure that can eliminate or significantly reduce animal trials [22] (https://www.epa.gov/sciencematters/epa-releases-updated-new-approach-methodologies-nams-work-plan, accessed on 23 May 2024), strongly reducing the time, the cost, and the ethical limitations required for the in vivo experiments. In particular, predictive analyses performed through computer aid are becoming increasingly popular, especially due to recent developments in artificial intelligence. Indeed, several NAMs have been performed to predict chemical toxicity on human health [23,24,25]; however, very few of them are related to the effects of chemicals on human reproduction [26,27]. The aim of this study is to clarify some of the possible effects at the molecular level of toxic compounds on human fertility through the prediction of their interactions with proteins involved in the reproduction pathways. For this purpose, we performed a computational study on the interaction between the 3D structures of several toxic chemicals widely distributed in the environment, such as pesticides and fertilizers, with the 3D structure of human proteins which play a significant role in human reproduction. The study is based on the hypothesis that, even if these molecules appear not to have specific or toxic action reported affecting human reproduction, they may instead interfere at the structural level with proteins involved in important physiological processes, causing disastrous effects on the fertilizing capacity of gametes.

Three datasets of 3D protein structures have been prepared, two referring to male and female gametes, and the third containing the proteins responsible for gamete fusion. The chemical molecules were instead obtained from the free online databases of available compounds, or prepared with specific modeling software. Molecular docking and virtual screening approaches were used to determine the binding energies and affinities between molecules (ligands) and proteins (receptors). In particular, the results predicted some interesting interactions between famoxadone with important proteins involved in the ovary, spermatozoon, and zygote formation. With the support of a thorough literature search of the available articles on the subject, new hypotheses about the mechanism of action and the effects that famoxadone might have on human fertility have been proposed.

## 2. Results and Discussion

### 2.1. Molecular Docking Analysis Using AutoDock Vina

**Figure 1:**
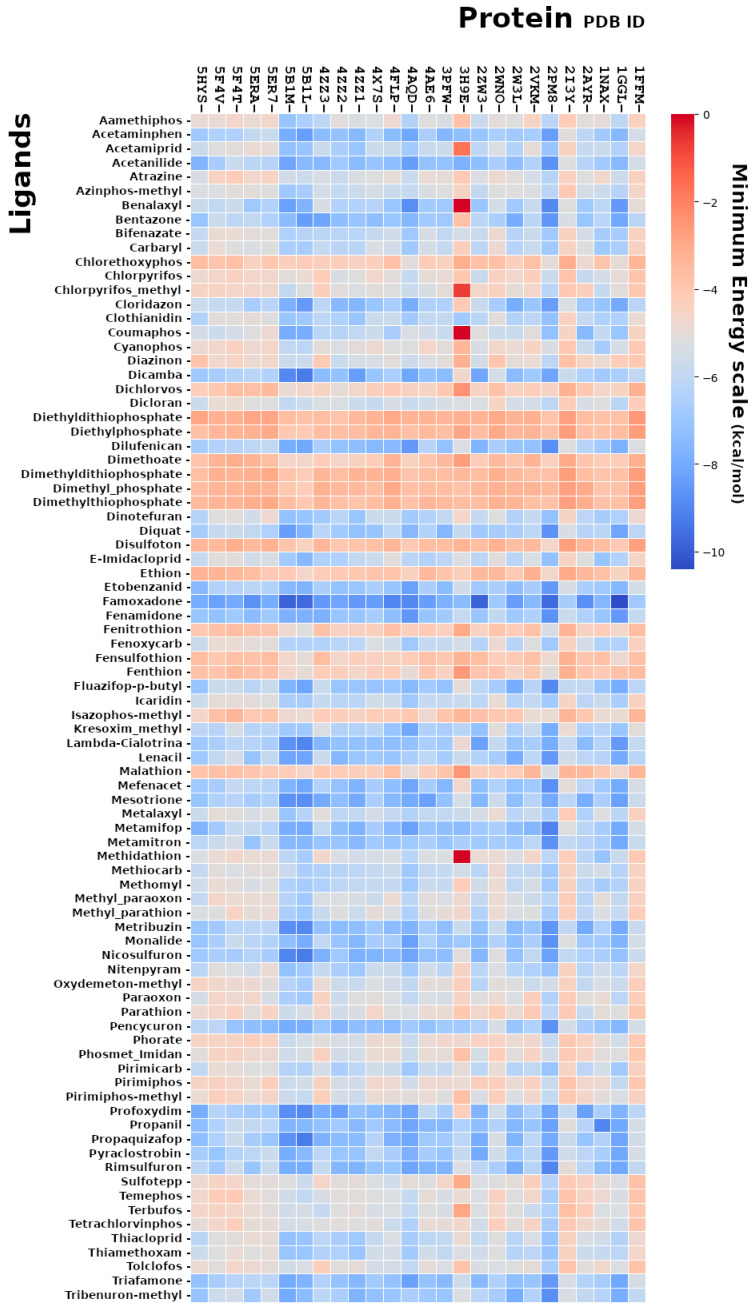
Heatmap representation of the predicted minimum energy values for the binding of the ligands in the whole ligands dataset against a number of proteins showing the more significant interactions.

Molecular docking is used to predict the preferred orientation of a molecule (ligand) towards a protein (receptor) and to allow the study of these interactions. We exploited this methodology in order to perform a “virtual screening” approach for the study of possible interactions between toxic compounds and proteins related to reproduction. We obtained over 70,000 poses describing the protein–ligand interactions by the virtual screening analysis, and, from the comparison of the minimum energy values, expressed in kcal/mol, we identified the toxic ligands predicted to bind to the proteins involved in the reproduction processes with a high affinity (Figure 1).

Among all the analyzed ligands in the dataset, famoxadone (Figure S1), a recent antiparasitic fungicide normally used in agriculture to prevent and treat diseases such as downy mildew (*Phytophthora infestans*), was predicted to interact with good affinity to a large number of proteins associated to human reproduction. It is reported that famoxadone controls plant pathogens through the inhibition of electrons transferred into the mitochondria of pathogens, resulting in their death due to the loss of ATP as an energy source [28]; thus, it should not be considered harmful for animal reproduction.

The proteins showing the higher affinity towards famoxadone in the range of minimum energy values from −10.0 to −8.0 kcal/mol included the cellular retinol binding protein-III (CRBP-III), the connexin-26 gap junction channel, the histone H3.1 in the nucleosome structure, the human butyrylcholinesterase and its glycosylated form, the bromodomain testis-specific protein (BRDT), the estrogen receptor, the human GAR transformylase, the anti-apoptotic Bcl-2 transcription factor, the sperm-specific isoform of Protein Kinase A (PKA), the IgE-Fc fragment, and the sperm–egg fusion protein 1 (IZUMO1) (Table S1).

The identified proteins play different roles in the male and female reproductive systems, so, excluding the antibodies and receptors, which could not specifically bind famoxadone, and the anti-apoptotic Bcl-2 transcription factor [29,30], as it is unrealistic to assign a famoxadone involvement on a complex phenomena like apoptosis only on the basis of a computer prediction, we analyzed the docking poses and the biological functions of the remaining proteins with an affinity value lower than −8.0 kcal/mol.

The nucleosome histone H3.1 and the connexin-26 in the gap junction channel belong to big macromolecular complexes with a high molecular weight and are difficult to associate to a direct influence of famoxadone on their structural function; moreover, there is no evidence in the literature of the effects of famoxadone on their functions. On the other hand, butyrylcholinesterase seems to bind almost all the compounds because of its similarity to Acetylcholinesterase, which is the main target of a number of pesticides. Thus, it becomes difficult to associate a predicted binding to the famoxadone to a specific effect on the enzyme function in the absence of experimental evidence, such as a measured irreversible inhibition. About BRDT, it is demonstrated that the inhibition of its function can cause a reversible reduction in the number and motility of mouse spermatozoa [31]. Similarly, the inhibition of the human GAR transformylase, which is a key enzyme at the initial steps of the de novo purine biosynthesis pathway for DNA replication [32], and of the PKA, which is demonstrated to be important for sperm motility [33], should affect the sperm number and motility, respectively. However, in the literature, none of these noticeable effects were reported for famoxadone.

Then, we focused our attention on two proteins, CRBP-III (PDB-ID 1GGL), having predicted the highest affinity toward famoxadone, and IZUMO1 (PDB-ID 5F4V), which is a key protein in the reproductive mechanism.

### 2.2. CRBP-III Structure, Sequence, and Functional Role

In particular, the CRBP-III protein plays a role in the ovary by binding in the cytosol the cellular retinol (or Vitamin A) [34], which is involved in several biological processes of the human body, including fertility [35]. A member of the CRBP superfamily (small monomeric proteins capable of hydrophobic ligand binding), along with CRBP-I and CRBP-II, with distinct tissue distributions and retinol-binding properties (binding retinol and not retinaldehyde [36]), CRBP-III is the member of the family that has been less characterized structurally. CRBP-III has a β-barrel structure, consisting of ten parallel β-sheets [37] (Figure S2), and shares about 50% identity of sequence with CRBP-I (55.6%) and II (49.6%). However, inside the binding site, the glutamine residue at position 108 in the CRBP-I and II sequences has been replaced by histidine residue in the CRBP-III sequence (Figure S3). This histidine residue was predicted to act both as a donor and as a hydrogen bond acceptor, stabilizing retinol binding through the formation of hydrogen bonds with the hydroxyl group of the retinol molecule [34]. From the comparison of the CRBP-III structure with the rat holo-CRBP II (PDB ID code 1OPB) structure bonded to retinol [34], other amino acid residues at position 20, 33, 36, 38, 40, 42, 53, 58, 77, 106, and 119 are predicted to contribute to the binding to retinol, together with histidine 108. In particular, the residues K40, T53, R58, and W106, which are within 3.6 Å of the retinol molecule in the holo-CRBP II, are highly conserved along the CRBPs [34].

### 2.3. CRBP-III–Famoxadone Interaction Model

**Figure 2:**
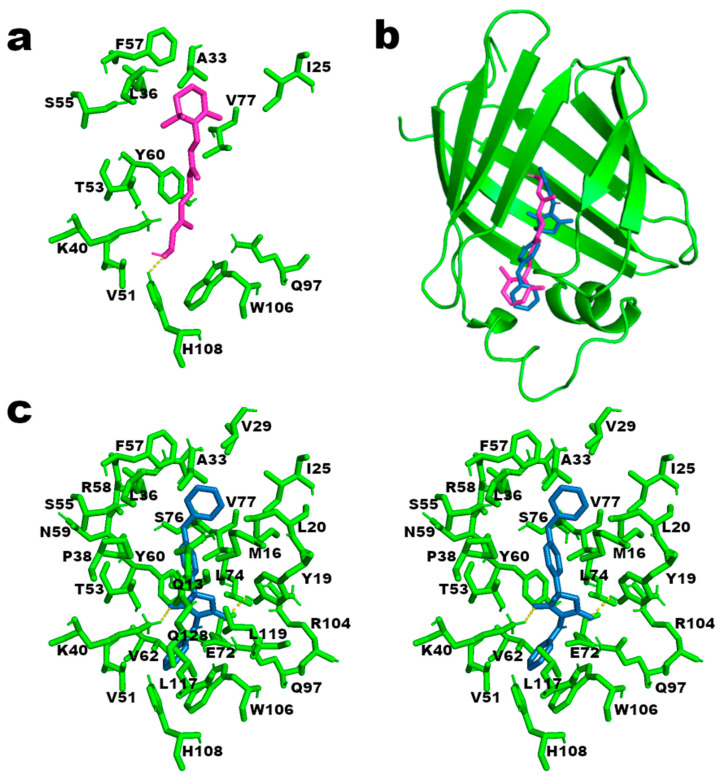
(**a**) Stick representation of CRBP-III amino acid network (in green) surrounding the retinol structure (purple) at a distance ≤ 4 Å. The hydrogen bond with H108 is in yellow. (**b**) A 3D representation of the CRBP-III structure (green, in cartoon format) binding retinol (purple, in stick format) and famoxadone (light blue, in stick format). (**c**) On the left, stick representation of CRBP-III amino acid network (in green) surrounding famoxadone (in light blue) at less than 4 Å. On the right, the same representation where residues Q13, L119, and Q128 are removed to permit a better visualization of the predicted hydrogen bonds (yellow) of residues Y19 and K40 with famoxadone.

In our molecular docking prediction using retinol as the ligand and the CRBP-III structure as the receptor, we found a similar network of amino acid residues surrounding the retinol molecule as predicted in the literature, in particular, residues A33, L36, K40, T53, V77, W106, and H108 (Figure 2a). In addition, we also found residues I25, V51, S55, F57, Y60, and Q97 in the proximity of the retinol molecule (<4 Å distance) (Figure 2a). The H108 hydrogen bonds with the hydroxyl group of the retinol is also confirmed using the find polar contacts command in the PyMOL ver 2.5.0 software.

From the molecular docking analysis using famoxadone as the ligand, we observed that the pesticide shares the same amino acid residue network interacting with retinol; indeed, from the superposition of the poses, both the ligands are located in the same binding cavity in the CRBP-III structure (Figure 2b and Figure S3) with an RMSD l.b. of 3.77 determined using the RMDS tool in VMD ver 1.9.4 [38]. However, the interaction of CRBP-III with famoxadone includes a larger number of amino acid residues with respect to retinol, and is further stabilized by some hydrophobic patches including L20, V29, P38, V62, L74, L117, and L119 amino acid residues (Figure 2c). Moreover, using the find polar contacts command of PyMOL software, hydrogen bonds between the Y19 and K40 side chains, with the two oxygens of the oxazolidinedione ring of famoxadone, are expected.

These observations find confirmation in the minimum energy value of −10.4 Kcal/mol determined for the best pose of famoxadone in the CRBP-III structure. This value is lower than the one of −7.1 Kcal/mol obtained by the docking analysis conducted using retinol as a ligand, indicating a more stable CRBP-III/famoxadone complex with respect to the CRBP-III/retinol one.

At present, famoxadone toxicity on the fertility and development of animals or even humans has been poorly studied, because the pesticide mechanism of action can be considered harmful for animal reproduction [39,40]. However, recent studies in the field of cardiovascular diseases conducted on zebrafish as a model organism, because of its highly similar cardiovascular system development and 87% similarity in genome with the human heart [41], have highlighted the induction of cardiotoxicity in the embryos of these organisms in the presence of famoxadone [40]. These studies demonstrated that, when exposure to different concentrations of the fungicide is extended up to 72 h post-fertilization, significant morphological changes in the heart and serious consequences for cardiac function are observed in embryos [39,40]. In addition, high levels of oxidative stress, uncontrolled proliferation of myocardial cells, apoptosis phenomena, and alterations in ATPase activity and in the expression of genes related to cardiac development and calcium pathway signaling have also been detected [40].

**Figure 3:**
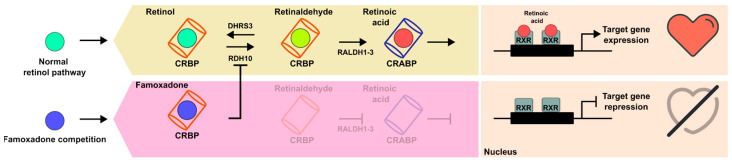
Hypothetical model of famoxadone interference in the normal retinol pathway through the predicted binding with CRBP-III.

Our predictions suggest a possible competition of famoxadone and retinol for the same binding site, and, considering the better affinity and the increased network of interactions stabilizing the pesticide binding, it appears that the CRBP-III complex with famoxadone is more favorable. For this reason, a low amount of famoxadone could be necessary to significantly interfere with the physiological binding of retinol to CRBP-III, as required in order to act as a competitive inhibitor. Moreover, these effects would be more marked during the tissue development of an embryo, because a chemical concentration accumulating in a small-sized organism, metabolically accelerated, even if low, is more significant with respect to the same concentration in an adult organism [42]. Due to the role played by CRBP-III during the embryonic cycle, these results allow us to hypothesize that the effects observed in zebrafish embryos exposed to famoxadone could be exercised through a competitive inhibition of CRBP-III, altering the normal metabolic cycle of retinol (Figure 3). Therefore, if experimentally demonstrated, famoxadone could cause, through the inhibition of CRBP-III functions, serious reproductive problems for fertility in adults, affecting embryo development even in the absence of physiological problems.

### 2.4. IZUMO1 Structure, Sequence, and Functional Role

The protein IZUMO1 [43,44], which has an important role in the sperm–oocyte fusion and is essential for male fertility [45], also appears to bind famoxadone with a good minimum energy value (in the range from −8.3 to −8.0 Kcal/mol). IZUMO1 is a monomeric type 1 transmembrane protein of 377 amino acids, with a broad mixed α/β secondary structural character [44,46]. The overall 3D structures available in the online database (232–246 residues) consists of two domains: a rod-shaped N-terminal with a 4-helical bundle (4HB) and a C-terminal bundled domain similar to that of immunoglobulin, connected by a β-hairpin (Figure S4).

About the role of IZUMO1, the N-terminal domain directs recognition, and adhesion occurs via multiple low-affinity interactions (Van der Waals, ionic, and hydrophobic interactions) with JUNO, a cysteine-rich glycoprotein anchored to glyco-phosphatidyl-inositol (GPI) that resides on the surface of the oolemma [43,44,47,48]. After contact has been established, a series of conformational changes follow within IZUMO1, which trigger the progression of the 4HB domain in proximity to the egg membrane [43,44,49]. In the next step, the fusion phase of the two plasma membranes occurs through the interaction of IZUMO1 with the surface protein CD9 [44,46]. Therefore, when the IZUMO1, JUNO, and CD9 proteins are missing, or their functions are altered, even if the spermatozoa and eggs produced are normal in number, appearance, and behavior, fertilization fails in the final phase of the adhesion and fusion of gametes [50,51,52].

### 2.5. IZUMO–Famoxadone Interaction Model

**Figure 4:**
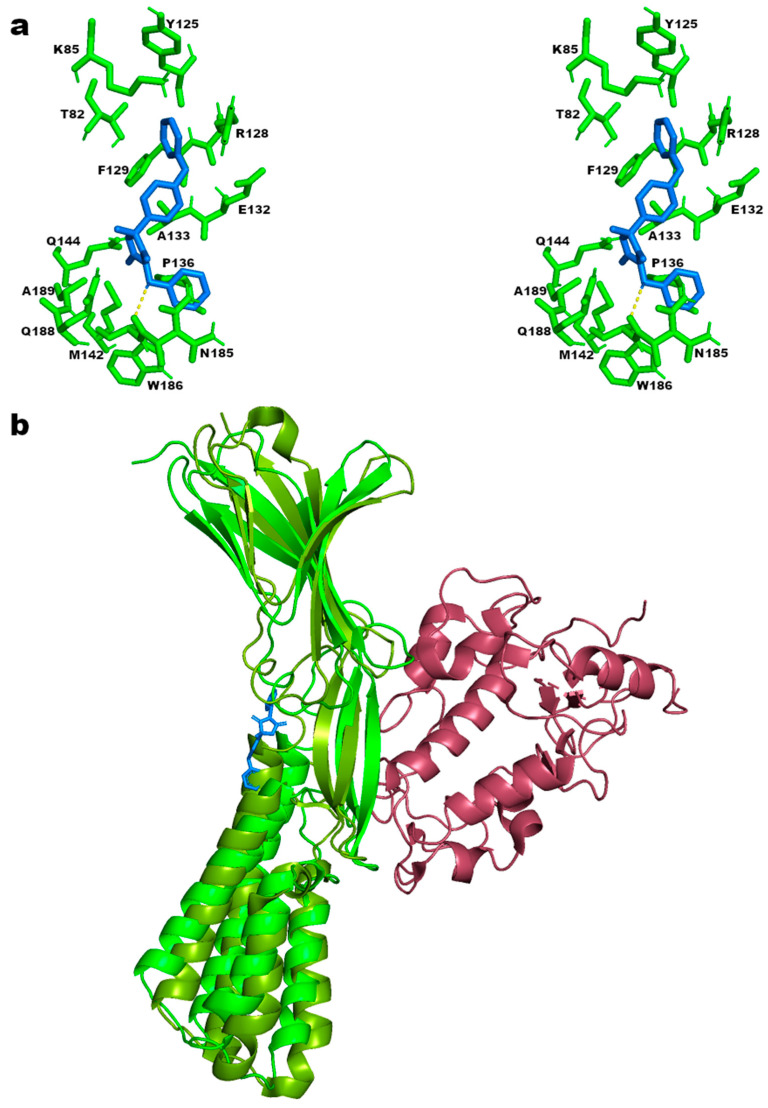
(**a**) Stereo view in stick representation of IZUMO1 (PDB-ID 5F4V) amino acid network (in green) surrounding the famoxadone structure (light blue) at a distance ≤ 4 Å. In yellow, the predicted hydrogen bond with the N185 residue. (**b**) A 3D representation of the famoxadone best-predicted pose on the IZUMO1 structure with respect to the binding surface of JUNO. The picture was obtained overlapping the IZUMO1 structure (PDB-ID 5F4V) (cartoon representation in green) used for the prediction of famoxadone (stick representation in blue) binding, with the IZUMO1 structure (cartoon representation in olive green) in complex with JUNO structure (cartoon representation in violet) (PDB-ID 5F4E). RMSD of the two IZUMO1 structures overlapping is 3.69, determined using the align tool in Pymol.

Unlike the interaction with CRBP-III, in which famoxadone enters the retinol-binding site and a competitive effect can be hypothesized, the interaction between IZUMO1 and famoxadone was predicted to occur on the surface of the protein (Figure S5). In particular, famoxadone binds between the 4HB and Ig-like domains of IZUMO1, interacting with a network of polar (T82, K85, Y125, R128, E132, Q144, N185, and Q188) and non-polar (F129, A133, P136, M142, W186, and A189) amino acid residues in the cavity between the two domains (Figure 4a). The binding in this pose is also more stabilized by a predicted hydrogen bond of the oxygen atom between the two phenolic rings of famoxadone and the N185 residue of IZUMO1 (Figure 4a, dashed line in yellow). Regarding a possible steric hindrance, it could be assumed that this binding has no significant impact on the JUNO recognition function of IZUMO1, because the binding occurs in an area opposite to the binding interface between IZUMO1 and JUNO (Figure 4b and Figure S6). This result suggests that there is no type of interference of the fungicide molecule in the interaction between the two proteins.

**Figure 5:**
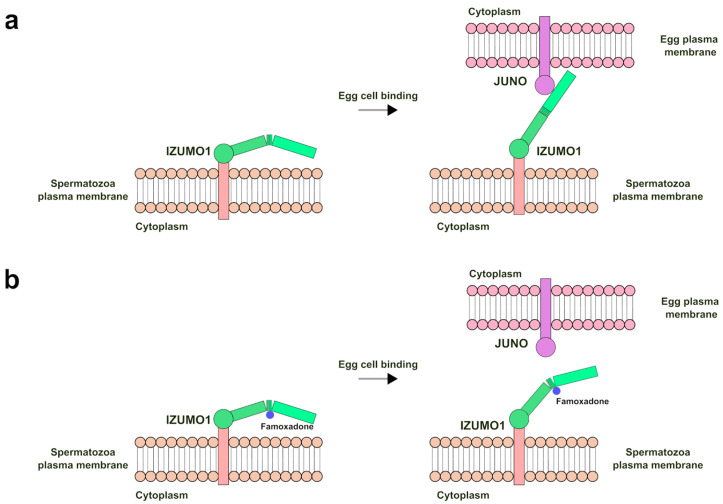
Hypothetical model of famoxadone interference in the IZUMO1-JUNO interaction: (**a**) IZUMO1-JUNO binding after IZUMO1 conformational change; and (**b**) interference of famoxadone on the IZUMO1-JUNO binding through disturbance of IZUMO1 conformational change.

However, from a recent work in the literature [49], it has been observed that the cavity between the two 4HB and Ig-like domains, involved in the famoxadone binding, undergoes conformational changes. These rearrangements are necessary for the binding with JUNO and the progression of the domain 4HB towards the egg membrane in order to allow subsequent membrane fusion (Figure 5a).

In accordance with these observations, a molecular docking analysis performed on the IZUMO1 structure in complex with JUNO (PDB-ID 5F4E) predicted a different spatial orientation of the famoxadone molecule in the cavity between the 4HB and Ig-like domains, with a lower affinity value of −7.148 kcal/mol with respect to the pose of IZUMO1 structure without JUNO (PDB-ID 5F4V). Moreover, the predicted pose involved a different network of amino acid residues; in particular, fewer new residues (D79, E80, T145, K154, A189, and E191) are involved in the binding, and only residues A133, P136, Q144, N185, and W186 are maintained, even if the hydrogen bond with N185 is absent (Figure S7). Therefore, the predicted high affinity, the presence of a hydrogen bond, and the larger number of amino acid residues around famoxadone in the pose of IZUMO1 structure without JUNO support the idea that the famoxadone molecule could stabilize this IZUMO1 structural conformation, interfering through a far steric hindrance with the IZUMO1-JUNO binding. In particular, it can be hypothesized that its interaction in the cavity between the 4HB and Ig-like domains could affect the two IZUMO “boomerang” and “straight” conformations identified by crystallography [49]. The failure in the rearrangement of the 4HB domain with respect to the Ig-like one could prevent the interaction between IZUMO1 and JUNO, interfering with the mechanism of membrane approximation and arresting the gamete fusion (Figure 5b).

Although it is extremely complicated to evaluate the effects of the fungicide on the conformational changes of the IZUMO1-JUNO macromolecular complex, further studies, such as molecular dynamics (MD), an in vitro protein–ligand binding assay, or a X-ray crystallographic analysis of the IZUMO1-famoxadone complex, are necessary to confirm the reliability of the predicted poses, in order to demonstrate this type of inhibition of the IZUMO function.

Similarly to CRBP-III, this kind of effect cannot be determined through the actual methodologies which just measure the numbers and motility of spermatozoa or the absence/presence of oocytes. This evidence highlighted the necessity to update the way to diagnose idiopathic infertility, through the use of molecular level investigations that permit identifying the real cause affecting fertility.

## 3. Materials and Methods

### 3.1. Ligand Dataset Preparation

A list of several chemicals belonging to different pesticide families, such as carbamates and organophosphates, are chosen on the basis of their widespread diffusion in the environment and their availability in 3D structure databases (Table S1 in Supplementary Materials). The datasets were prepared by several databases, such as ZINC, a free database of commercially-available compounds for virtual screening (https://zinc15.docking.org/, accessed on 23 May 2024), and PubChem, an open chemistry database at the National Institutes of Health (NIH) (https://pubchem.ncbi.nlm.nih.gov/, accessed on 23 May 2024). Avogadro 1.2.0 software, a free advanced editor and molecule visualizer designed for the use of molecular modeling in bioinformatics, was used for preparation of some ligands. Using Simplified Molecular Input Line Entry System (SMILES), which is a representation of chemical structure by means of text strings in ASCII format, as an input format in Avogadro, it was possible to generate 3D chemical structures of molecules not available in the online databases, and optimize their molecular structure by applying different force fields (https://avogadro.cc/, accessed on 23 May 2024). The SMILES was obtained from the ZINC and PubChem databases and the structures were optimized by applying the force field MMFF94 (Merck Molecular Force Field) specifically parameterized for organic compounds, with a maximum slope, until the dE was ≤0.01. The 3D structures obtained in this way were exported as “.pdb” file format necessary for the subsequent studies of molecular docking.

### 3.2. Protein Datasets Preparation

Three datasets of 3D protein structures referring to male and female gametes, and for gamete fusion, have been prepared (Table S2 in Supplementary Materials). The datasets included 68, 39, and 13 structures belonging to 33, 15, and 3 proteins of the sperm, ovary, and fusion datasets, respectively (Tables S3 and S4 in Supplementary Materials). The protein structure files in “.pdb” format are obtained from the RCSB-PDB database, a global Protein Data Bank (PDB) archive of 3D structure data for large biological molecules (proteins, DNA, and RNA) (https://www.rcsb.org/, accessed on 23 May 2024). Using the software Pymol ver. 2.5.0 (https://pymol.org/2/, accessed on 23 May 2024), the structures were checked for breaks in the amino acid sequence, individual subunits were extracted from the protein complexes/oligomers, and the heteroatoms, such as water molecules and any residual non-standard ligand, were removed such that the entire surface of the proteins were available to the binding with the ligands of interest.

### 3.3. Molecular Docking Analysis

The molecular docking was performed on the datasets of proteins and ligands previously prepared. For molecular docking simulations, it is important to prepare input files of both ligands and receptors. For this purpose, AutoDockTools ver. 1.5.7 (ADT) was used (http://mgltools.scripps.edu/, accessed on 23 May 2024), a docking support software designed for structure setup and analysis of docking results. Using ADT, it was possible to add the polar hydrogens to the protein structures verified with Pymol and set the information on the torsion angles of the ligands. During the docking procedure, the proteins are considered rigid, because of the blind search for a binding site and to reduce the analysis time, while ligands are permitted to rotate on the torsion angles as determined in ADT software. All structures (proteins and ligands) were exported in the “.pdbqt” format required by the molecular docking software.

In addition, for each protein in our dataset, a grid box was drawn, representing the actual volume within which the ligand can move, whose dimensions were defined in such a way as to optimally encompass the possible binding sites of the protein. In particular, in order to perform a blind molecular docking and to permit the ligand molecules to explore freely the proteins surface and cavities, binding all the possible sites, we made grid boxes incorporating the proteins with a margin of 1.5 Å from the surface. For each protein structure was prepared a configuration file ‘config.txt’, containing the spatial coordinates X, Y, and Z obtained by drawing the grid box, and setting parameters of the “exhaustiveness”, representing the time spent searching for the energy minimum of the protein–ligand conformations. The value of “exhaustiveness” used was only doubled to 16, as, while linearly increasing, the analysis time decreased exponentially the probability of not finding the best minimum global minimum of binding energy.

The AutoDock Vina ver 1.1.2 software (http://vina.scripps.edu/, accessed on 23 May 2024), an open-source programme for molecular docking receptor-binding, was used for the docking analysis [53]. AutoDock Vina represents a good choice because it is a good compromise between precision and speed of analysis, uses the “.pdbqt” format exported by ADT, and, in addition, offers the possibility of performing docking analysis on protein and ligand datasets through the use of scripts to automate their screening.

Indeed, a “virtual screening” approach was used for the molecular docking analysis. In fact, the manual analysis of thousand protein–ligand interactions (about 120 protein structures and 82 ligand structures for over 70,000 poses) becomes too time-consuming. It was, therefore, necessary to automate the entire docking analysis process, through the integration with shell scripts on a Linux OS platform, to complete the entire proposed study. Specifically, the script (in Supplementary Materials) allowing the docking analysis in an automated manner is programmed in Linux Bash Shell language. The script is started in the work folder where the structure files are located, which have been renamed “protein_name.pdbqt” and “ligand_name.pdbqt” for proteins and ligands, respectively, where “name” represents the PDB ID of the protein or the name of the ligand. The script calls up the Vina software by passing it the structure file of the first protein and the first ligand, together with the configuration information contained in the “config” file of the protein. At the end of the analysis with the first ligand, the script restarted with the second ligand, and then the third, and so on. At the end of the analysis on the first protein with all the ligand files contained in the folder, the script calls up the second protein together with its configuration file and restarts with the analysis of the first ligand. The script terminates when all proteins have been analyzed by Vina.

The output of Vina consists in a file containing 8 or more poses (the spatial co-ordinates of the ligands) for each protein–ligand interaction. Through the script, another file is generated, containing the information related to the RMSDs and minimum energy values of the complex for each pose, which represents a single interaction between the protein and the ligand, expressed in Kcal/mol. The lower this value, the greater the affinity of the ligand towards the protein.

Finally, each pose of the structural models that showed minimum energy values (high affinity) were visualized using Pymol to confirm the presence of interactions and compared with the same structures containing other ligands, if available.

### 3.4. Bibliography Research

A cross-research on the PubMed database (https://pubmed.ncbi.nlm.nih.gov/, accessed on 23 May 2024) was performed in order to identify the involvement of famoxadone with the proteins identified by the virtual screening analysis. The search stream used was famoxadone* OR human* OR reproduction* OR fertilit* OR sperm* OR oocyte* OR ovar* OR (name_of_the_protein_or_abbreviation). The information obtained was used for the selection of the protein to analyze and to support the description of the possible mechanism of action of the famoxadone on human reproduction.

## 4. Conclusions

Reproductive health represents a very important aspect of human health and in general for the living organisms. Therefore, it is necessary to improve the knowledge about the possible causes affecting fertility, in particular, the causes underlying the idiopathic infertility, which still remain unknown. Considering the increased number of external factors, such as chemicals to which we are exposed every day through food, air, and water, computer-assisted approaches can help to identify new interactions at the molecular level and to address the studies in a more specific way. This aspect is particularly important for the studies on human fertility, because, in addition to the excessive effort required to perform experiments on a large number of chemicals, the analyses on reproductive tissues and cells are hampered by experimental and legislative limitations. Through a virtual screening approach using molecular docking conducted on datasets of reproduction-related protein structures and potentially toxic ligands, we demonstrated the possibility to obtain information on some predicted interactions that could affect human reproductive health. In particular, our results suggested that famoxadone residues, which could be present in products deriving from agricultural crops or in water, may be harmful for human reproductive health. Moreover, its predicted binding with proteins involved in the formation and development of the zygote permits hypothesizing some functional models of interaction that are able to explain some experimental evidence obtained on a model organism such as zebrafish. Moreover, the effects that we hypothesized on the functions of the target proteins are usually not taken into account in the ordinary toxicity assessment. In perspective, the addition of molecular dynamics studies on the protein–ligands complexes predicted by molecular docking, in order to confirm the reliability of the predicted poses, will help to support the hypothesized mechanism of action, particularly when conformational changes are involved as for the IZUMO1 “boomerang” and “straight” conformations.

Therefore, our approach, or similar approaches that are bioinformatic-based, can be useful in improving the information about the reproductive health risk of chemicals and the possibility of directing the subsequent in vitro and in vivo testing in a more specific manner, reducing the cost and the time required for the validation of the study.

In conclusion, this study remains only the first step towards understanding the effects of some ligands, whose toxicity at certain concentrations and in specific target organs is not yet clear.

## Data Availability

The data presented in this study are available on request from the corresponding author.

## Supplementary Materials

The supporting information can be downloaded at: https://www.mdpi.com/article/10.3390/ijms25115790/s1.
