# Supplementary material for: Prediction of Pesticide Interactions with Proteins Involved in Human Reproduction by Using a Virtual Screening Approach: A Case Study of Famoxadone Binding CRBP-III and Izumo
Source: Int J Mol Sci. 2024 May 26;25(11):5790. doi: 10.3390/ijms25115790 (PMC11171824; doi:10.3390/ijms25115790)
Supplement: Supplementary file 1 [file ijms-25-05790-s001.zip › ijms-3023845-supplementary.pdf]

Prediction of Pesticide Interactions with Proteins Involved in Human Reproduction by Using a Virtual Screening Approach: A Case Study of Famoxadone Binding CRBP-III and Izumo

Fabiana Tortora [1,2], Valentina Guerrera [2], Gennaro Lettieri [3], Ferdinando Febbraio [2,*] and Marina Piscopo [3]

[1] Institute of Genetics and Biophysics “Adriano Buzzati Traverso”, National Research Council (CNR), via P. Castellino 111, 80131 Naples, Italy; fabiana.tortora@igb.cnr.it (F.T.)

[2] Institute of Biochemistry and Cell Biology, National Research Council (CNR), via P. Castellino 111, 80131 Naples, Italy; valentina.guerrera@ibbc.cnr.it (V.G.), ferdinando.febbraio@cnr.it (F.F.).

[3] Department of Biology, University of Naples Federico II, Via Cinthia, 21, 80126 Naples, Italy; gennarole@outlook.com (G.L.); marina.piscopo@unina.it (M.P.)

*Correspondence: ferdinando.febbraio@cnr.it; Tel.: +39 081 6132 611

Abstract: In recent years, the awareness that pesticides can have other effects than generic toxicity, is growing. In particular, several pieces of evidence highlight their influence on human fertility. In this study, we investigated by a virtual screening approach the binding between pesticides and proteins present in human gametes or associated to reproduction, in order to identify new interactions that could affect human fertility. To this aim, we prepared ligands (pesticides) and receptors (proteins) 3D structure datasets from online structural databases (such as PubChem and RCSB), and performed a virtual screening analysis using Autodock vina. In the comparison of the predicted interactions, we found that famoxadone was predicted to bind Cellular Retinol Binding Protein-III in the retinol binding site with a better minimum energy value of -10.4 Kcal/mol and an RMSD of 3.77 with respect to retinol (-7.1 Kcal/mol). In addition to a similar network of interactions, famoxadone binding is more stabilized by additional hydrophobic patches including L20, V29, A33, F57, L117 and L118 amino acid residues and hydrogen bonds with Y19 and K40. These results support a possible competitive effect of famoxadone on retinol binding with impacts on the ability of developing the cardiac tissue, as in accordance with literature data on zebrafish embryos. Also, famoxadone binds, with a minimum energy value between -8.3 and -8.0 Kcal/mol, to the IZUMO Sperm Egg Fusion Protein interacting with a network of polar and hydrophobic amino acid residues in the cavity between the 4HB and Ig-like domains. This binding is more stabilized by a predicted hydrogen bond with the N185 residue of the protein. An hindrance in this position can probably affect the conformational change for JUNO binding avoiding the gamete membrane fusion to form the zygote. This work opens new interesting perspectives of study on the effects of pesticides on fertility, extending the knowledge to other typology of interaction which can affect different steps of the reproductive process.

Keywords: Human reproduction; Molecular docking; Virtual screening; Pesticides; Cellular Retinol Binding Protein-III; IZUMO Sperm Egg Fusion Protein; Famoxadone

Script for virtual screening automation.

vina_script.sh

```
#! /bin/bash

# extract the protein PDB-ID from "protein_PBD-ID.pdbqt" namefiles
for p in protein_*.pdbqt; do
    c=`basename $p .pdbqt`
    d=${c##*_}
# extract the ligand name from "ligand_name.pdbqt" namefiles
  for f in ligand_*.pdbqt; do
    b=`basename $f .pdbqt`
    e=${b##*_}
    echo
    echo
    echo ANALYSIS
    echo Processing $c
    echo Processing $b
# (vina --config $c.config --receptor $p --ligand $f) = call the program autodock vina passing the config file,
receptor and ligand. (--out $c\_$b\_out.pdbqt) = save the output (poses) with a filename including ligand and
protein PDB-ID. ( >> docking_results.txt) append the vina output (affinity)in the file "docking_results.txt".
    vina --config $c.config --receptor $p --ligand $f --out $d\_$e\_out.pdbqt >> docking_results.txt
  done
done
```

Table S1. List of the proteins with predicted high affinity towards famoxadone in the range from -10 to -8 kcal/mol.

Dataset name	Protein names	Affinity (kcal/mol)
Sperm/epididymis proteins	connexin-26 gap junction channel	-9.8
	Histone H3.1 in nucleosome structure	-9.8 and -9.7
	Human bromodomain testis-specific protein (BRDT)	-9.0
	IgE-Fc fragment	-8.4
	Human Sperm-Specific Isoform of Protein Kinase A (PKA)	-8.4
Oocyte/ovary proteins	human cellular retinol binding protein III	-10.4
	human butyrylcholinesterase	-9.7 and -8.9
	estrogen receptors alpha and beta	-8.8
	human GAR transformylase	-8.5 and -8.2
	Anti-apoptotic Bcl-2	-8.4
Fusion proteins	Izumo1	-8.3 and -8.0

Table S2. List of chemicals used in the study.

Name	Class	Chemical formula
**Aromatic organic nitrogens**		
Benalaxyl	Fungicide	$C_{20}H_{23}NO_3$
Dicloran	Fungicide	$CL_2C_6H_2(NO_2)NH_2$
Famoxadone	Fungicide	$C_{22}H_{18}N_2O_4$
Fenamidone	Fungicide	$C_{17}H_{17}N_3OS$
Kresoxim-Methyl	Fungicide	$C_{18}H_{19}NO_4$
Metalaxyl	Fungicide	$C_{15}H_{21}NO_4$
Pencycuron	Fungicide	$C_{19}H_{21}CIN_2O$
Pyraclostrobin	Fungicide	$C_{19}H_{18}CIN_3O_4$
**Geodisinfestants**		
Dicamba	Herbicide	$C_8H_6Cl_2O_3$
Lambda-Cialotrina	Insecticide	$C_{23}H_{19}CIF_3NO_3$
Mesotrione	Herbicide	$C_{14}H_{13}NO_7S$
Metribuzin	Herbicide	$C_8H_{14}N_4OS$
Nicosulfuron	Herbicide	$C_{15}H_{18}N_6O_6S$
Profoxydim	Herbicide	$C_{24}H_{32}CINO_4S$
Propaquizafop	Herbicide	$C_{22}H_{22}CIN_3O_5$
**Anilides**		
Acetaminophen	Drug	$C_8H_9NO_2$
Acetanilide	Drug	C_8H_9NO
Diflufenican	Herbicide	$C1_9H_{11}F_5NO_2$
Etobenzanid	Herbicide	$C_{16}H_{15}CL_2NO_3$
Mefenacet	Herbicide	$C_{16}H_{14}N_2O_2S$
Metamifop	Herbicide	$C_{23}H_{18}CIFN_2O_4$
Monalide	Herbicide	$C_{13}H_{18}CINO$
Propanyl	Herbicide	$C_9H_9CL_2NO$
Triafamone	Herbicide	$C_{14}H_{13}F_3N_4O_5S$
**Heterocyclic organic nitrogens**		
Atrazine	Herbicide	$C_8H_{14}CIN_5$
Bentazone	Herbicide	$C_{10}H_{12}N_2O_3S$
Chloridazon	Herbicide	$C_{10}H_8CIN_3O$
Diquat	Herbicide	$C_{12}H_{12}Br_2N_2$
Fluazifop-P-Butyl	Herbicide	$C_{19}H_{20}F_3NO_4$
Lenacil	Herbicide	$C_{13}H_{18}N_2O_2$
Metamitron	Herbicide	$C_{10}H_{10}N_4O$
Rimsulfuron	Herbicide	$C_{14}H_{17}N_4O_7S_2$
Tribenuron-Methyl	Herbicide	$C_{15}H_{17}N_5O_6S$

**Carbamates**		
Bifenazate	Acaricide	$C_{17}H_{20}N_2O_3$
Carbaryl	Insecticide	$C_{12}H_{11}NO_2$
Fenoxycarb	Insecticide	$C_{17}H_{19}NO_4$
Icaridin	Repellent	$C_{12}H_{23}NO_3$
Methiocarb	Acaricide	$C_{11}H_{15}NO_2S$
Methomyl	Insecticide	$C_5H_{10}N_2O_2S$
Pirimicarb	Insecticide	$C_{11}H_{18}N_4O_2$
**Neonicotinoids**		
(E)-Imidacloprid	Insecticide	$C_9H_{10}CIN_5O_2$
Acetamiprid	Insecticide	$C_9H_{10}CIN_4$
Clothianidin	Insecticide	$C_6N_5H_8SO_2CL$
Dinotefuran	Insecticide	$C_7H_{14}N_4O_3$
Nitenpyram	Insecticide	$C_{11}H_{15}CIN_4O_2$
Thiacloprid	Insecticide	$C_{10}H_9CIN_4S$
Thiamethoxam	Insecticide	$C_8H_{10}CIN_5O_3S$
**Organophosphates**		
Azamethiphos	Insecticide	$C_9H_{10}CIN_2O_5PS$
Azinphos-Methyl	Insecticide	$C_{10}PN_3H_{12}S_2O_3$
Chlorethoxyfos	Insecticide	$C_6H_{11}Cl_4O_3PS$
Chlorpyrifos	Insecticide	$C_9H_{11}Cl_3NO_3PS$
Chlorpyrifos-Methyl	Insecticide	$C_7H_7Cl_3NO_3PS$
Coumaphos	Insecticide	$C_{14}H_{16}ClO_5PS$
Diazinon	Insecticide	$C_{12}H_{21}N_2O_3PS$
Dichlorvos	Insecticide	$C_4H_7Cl_2O_4P$
Disulfoton	Insecticide	$C_8H_{19}O_2PS_3$
Ethion	Insecticide	$C_9H_{22}O_4P_2S_4$
Dimethoate	Insecticide	$C_5H_{12}NO_3PS_2$
Diethylphosphate	Insecticide	$C_4H_{11}O_4P$
Dimethyl Phosphate	Insecticide	$C_2H_7O_4P$
Dimethyldithiophosphate	Insecticide	$C_2H_7O_2PS_2$
Dimethylthiophosphate	Insecticide	$C_2H_7O_3PS$
Fenitrothion	Insecticide	$C_9H_{12}NO_5PS$
Fensulfothion	Insecticide/Nematicide	$C_{11}H_{17}O_4PS_2$
Fenthion	Insecticide	$C_{10}H_{15}O_3PS_2$
Isazophos-Methyl	Insecticide	$C_7H_{13}CIN_3O_3PS$
Malathion	Insecticide/Acaricide	$C_{10}H_{19}O_6PS_2$
Methidathion	Insecticide	$C_6H_{11}N_2O_4PS_3$
Methyl-Paraoxon	Drug	$C_8H_{10}NO_6P$
Methyl-Parathion	Insecticide	$C_8H_{10}NO_5PS$

Oxydemeton-Methyl	Insecticide	$C_6H_{15}O_4PS_2$
Paraoxon	Oxon	$C_{10}H_{14}NO_6P$
Parathion	Insecticide/Acaricide	$C_{10}H_{14}NO_5PS$
Phorate	Insecticide/Acaricide	$C_7H_{17}O_2PS_3$
Phosmet (Imidan)	Insecticide	$C_{11}H_{12}NO_4PS_2$
Pirimiphos	Insecticide	$C_{11}H_{20}N_3O_3PS$
Pirimiphos-Methyl	Insecticide	$C_{11}H_{20}N_3O_3PS$
Sulfotep	Pesticide	$C_8H_{20}O_5P_2S_2$
Temephos	Larvicide	$C_{16}H_{20}O_6P_2S_3$
Terbufos	Insecticide/Nematicide	$C_9H_{21}O_2PS_3$
Tetrachlorvinphos	Insecticide	$C_{10}H_9Cl_4O_4P$
Tolclofos	Fungicide	$C_6H_{11}Cl_2O_3PS$

Table S3. List of the PDB ID entry in the three datasets, corresponding to the 3D protein structures used in the study.

**Dataset name**	**Protein PDB-ID**
Sperm/epididymis proteins*	1FPR - 1GWZ - 1X6C - 2B3O - 2I3Y - 2LL2 - 2RMX - 2ZW3 - 2YU7 - 3CYY - 3IZ1 - 3IZ2 - 3PS5 - 3H9E - 3PFW - 4AE6 - 4AE9 - 4FLP - 4GRY - 4GRZ - 4GS0 - 4HJP - 4HJQ - 4URT - 4NWY - 4X1L - 4X1M - 4X7S - 4X25 - 5B1L - 5B1M - 5C7L - 5C7O - 5CL7 - 5ER7 - 5ERA - 5HYS - 5KJ3 - 5KJG- 5KWY - 5WY0 - 5XUP - 6DKJ - 6FKQ - 6IIW - 6M90 - 6M91 - 6M92 - 6M93 - 6M94 - 6MXR - 6MXS - 6MY4 - 6MY5 - 6NAS - 6NBE - 6NBW - 6TTU - 6U1N - 6UVR - 6UVS - 6UVT - 6VEC - 6W5V - 6Z1N - 7NDG - 7NE0 - 7NE1
Oocyte/ovary proteins	1F7E - 1F7M - 1FF7 - 1FFM - 1GGL - 1NAV - 1NAX - 2AYR - 2J6L - 2PM8 - 2VKM - 2W3L - 2WKL - 2WNO - 3ALA - 4AQ3 - 4AQD - 4IEH - 4LVT - 4LXD - 4MAN - 4ZZ1 - 4ZZ2 - 4ZZ3 - 5AGW - 5IZQ - 5J9F - 6GL8 - 6KTC - 6KUG - 6O0K - 6O0L - 6O0M - 6O0O - 6O0P - 6QGG - 6QGH - 6QGJ - 6QGK
Fusion proteins*	5B5K - 5EJN - 5F4E - 5F4Q - 5F4T - 5F4V - 5JK9 - 5JKA - 5JKB - 5JKC - 5JKD - 5JKE - 5JYJ

*Some of the PDB structures belonging to these dataset contain protein complexes from which the individual polypeptide chains used for the analysis were extracted.

Table S4. List of the proteins in the three datasets used in the study.

Dataset name	Protein names	Classification
Sperm/epididymis proteins	protein-tyrosine phosphatase SHP-1	SIGNALING PROTEIN/HYDROLASE
	human glutathione peroxidase 5	OXIDOREDUCTASE
	connexin43	MEMBRANE PROTEIN
	connexin-26 gap junction channel	CELL ADHESION/MEMBRANE PROTEIN
	ZO-1 PDZ2 domain scaffold proteins	PEPTIDE BINDING PROTEIN
	human sperm-specific glyceraldehyde- 3-phosphate dehydrogenase apo/holo	OXIDOREDUCTASE
	Human Sperm-Specific Isoform of Protein Kinase A (PKA)	TRANSFERASE
	Human bromodomain testis-specific protein (BRDT)	TRANSCRIPTION REGULATOR
	Human netrin-1	PROTEIN BINDING/SIGNALING PROTEIN
	Deleted in Colorectal Cancer (DCC) receptor	PROTEIN BINDING
	disulfide isomerase-like protein of the testis (PDILT)	ISOMERASE
	profilin 1 (PFN1)	PROTEIN BINDING
	IgE-Fc fragment	IMMUNE SYSTEM
	Histone H3.1 in nucleosome structure	STRUCTURAL PROTEIN
	Alkylpurine DNA glycosylase AlkD	HYDROLASE
	Niemann-Pick disease type C1 membrane protein (NPC1)	TRANSPORT PROTEIN
	Niemann-Pick disease type C2 soluble protein (NPC2)	TRANSPORT PROTEIN

	Hua enhancer 1 (Hen1) 2'- *O* -methyltransferase	TRANSFERASE
	Telomere-binding protein TERB1	DNA BINDING PROTEIN
	Telomere shelterin subunit TRF1	DNA BINDING PROTEIN
	Glucose-dependent insulinotropic polypeptide (GIP) receptor (GIPR)	SIGNALING PROTEIN
	chromatin-binding E3 ubiquitin ligase ubiquitin-like with *PHD* and RING *finger* domains 1 (*UHRF1*)	LIGASE
	beta-*Trcp* (beta-Transducin repeat containing protein),	LIGASE
	S-phase kinase-associated protein 1	LIGASE
	dimeric bH1-Fab variant	IMMUNE SYSTEM
	N-alpha-acetyltransferase 80	TRANSFERASE
	E2 ubiquitin-conjugating enzyme UBE2D	LIGASE
	β-arrestin 1 (βarr1)	SIGNALING PROTEIN/IMMUNE SYSTEM
	M2 muscarinic receptor (M2R)	SIGNALING PROTEIN/IMMUNE SYSTEM
	actin-bundling protein plastin 3 (PLS3)	PROTEIN FIBRIL
	human heterotetrameric cis-prenyltransferase	TRANSFERASE
	Neogenin (NEO1)	SIGNALING PROTEIN
	repulsive guidance molecule B (RGM)	SIGNALING PROTEIN
Oocyte/ovary proteins	EGF-like domain from human blood coagulation FVII	BLOOD CLOTTING
	human cellular retinol binding protein III	TRANSPORT PROTEIN

	Thyroid Receptor Alpha	MEMBRANE PROTEIN
	Thyroid receptor beta1	MEMBRANE PROTEIN
	estrogen receptors alpha and beta	TRANSCRIPTION
	aminoadipate-semialdehyde dehydrogenase	OXIDOREDUCTASE
	human butyrylcholinesterase	HYDROLASE
	Anti-apoptotic Bcl-2	APOPTOSIS
	alpha acid-beta-glucosidase	HYDROLASE
	TNF-stimulated gene-6	CELL ADHESION
	BACE (Beta-secretase)	HYDROLASE
	human vascular adhesion protein 1	OXIDOREDUCTASE
	human GAR transformylase	TRANSFERASE/LIGASE
	human folate receptor alpha	SIGNALING PROTEIN
	human Y box binding protein 1	RNA BINDING PROTEIN
Fusion proteins	Izumo1	CELL ADHESION
	Juno	CELL ADHESION
	Izumo1 and Juno complex	CELL ADHESION

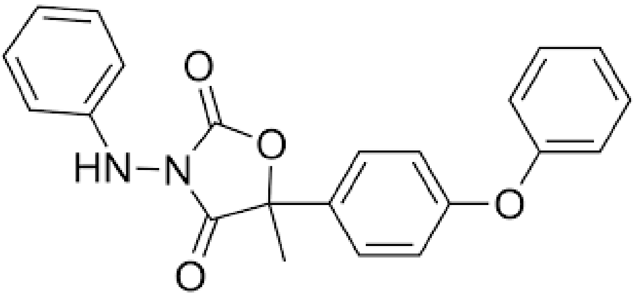

Figure S1. Chemical structure of famoxadone.

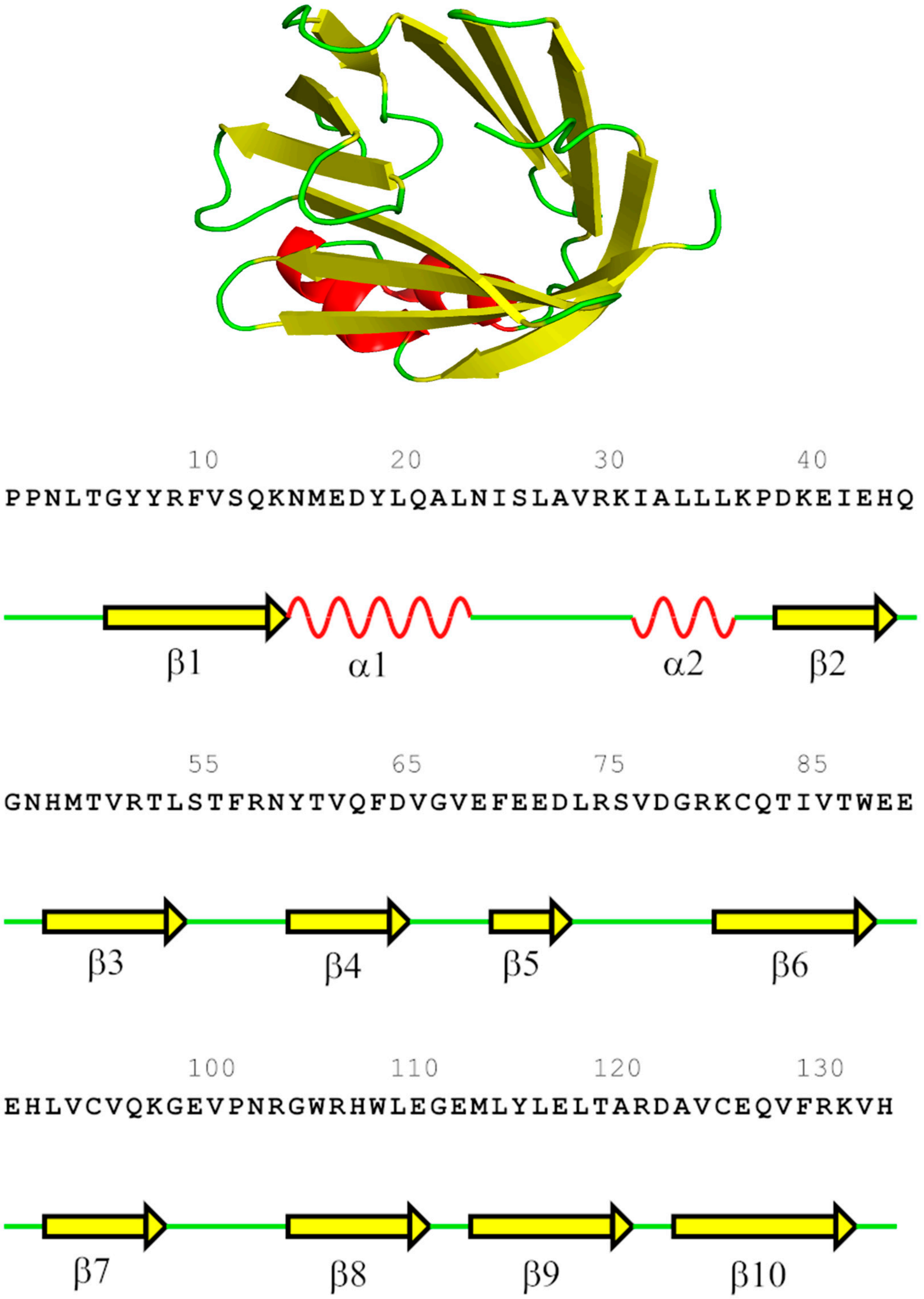

Figure S2. 3D representation (upper) and sequence, with 2D secondary structure representation (lower), of CBRP-III, indicating the β-barrel structure of the 10 β-sheets (in yellow) and the two α-helices (in red).

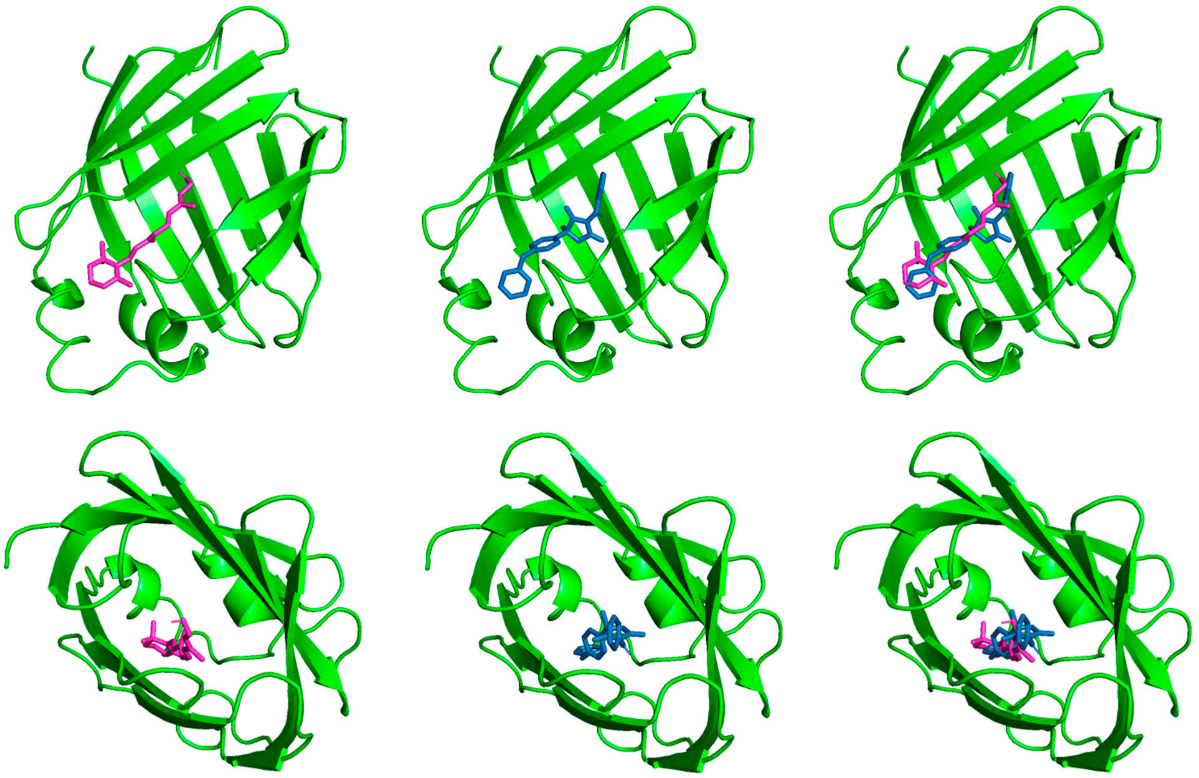

Figure S3. 3D representation of the structure of CRBP-III (green, in cartoon format) bound from left to right to the retinol (purple, in stick format), famoxadone (light blue, in stick format), and both ligands. Lower the same structures are rotated 90° with respect to the Y axis.

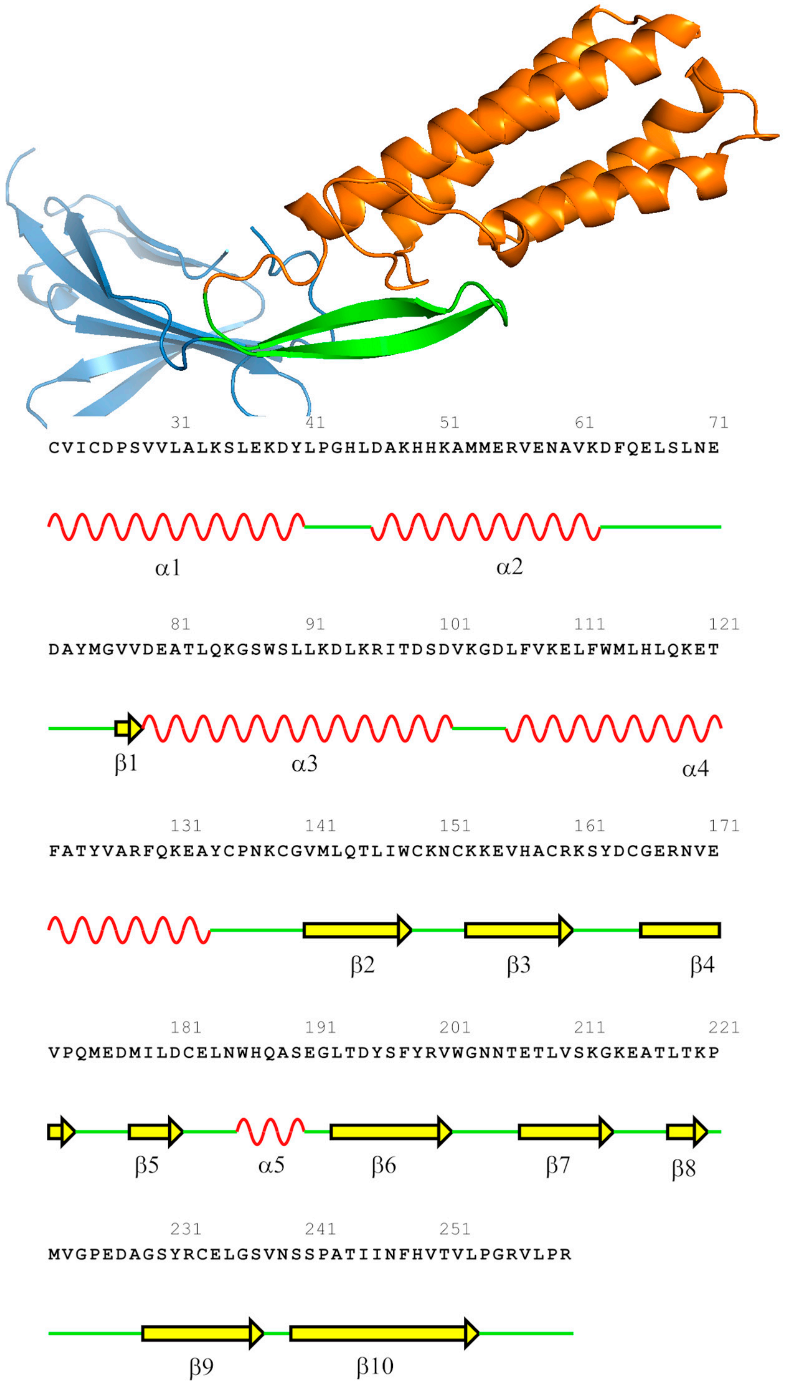

Figure S4. Cartoon representation (upper) of the α/β secondary IZUMO1 structure, the rod-shaped N-terminal domain with a 4-helical bundle (4HB) is in orange, the β-hairpin in green and the C-terminal Ig-like domain residues are in blue. Sequence and 2D secondary structure representation of IZUMO1 (lower), the β-sheets and the α-helices are in yellow and red, respectively.

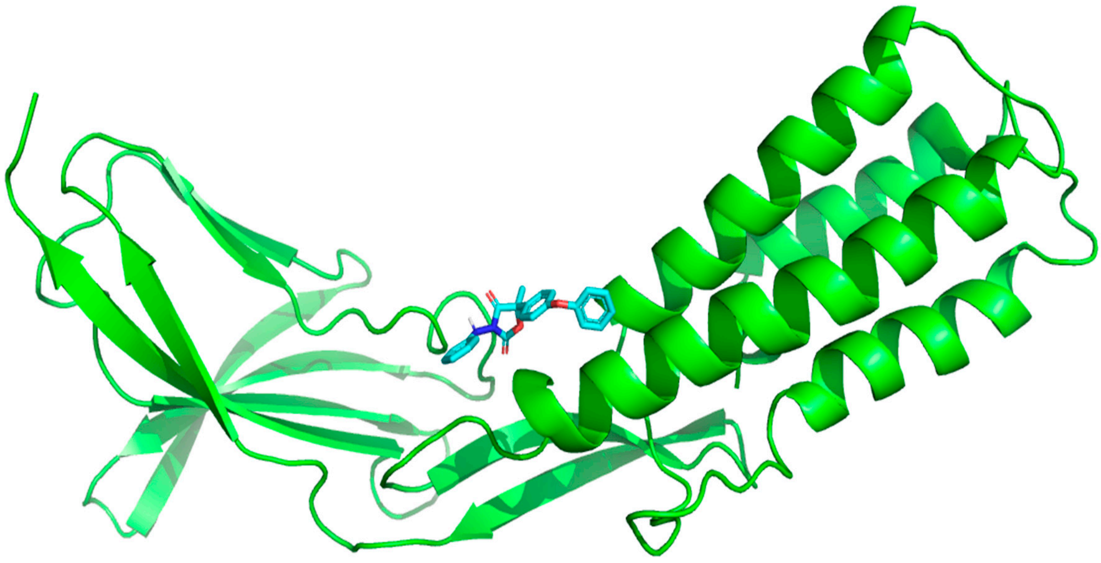

Figure S5. Cartoon representation of IZUMO structure (green) bound to famoxadone (in stick format).

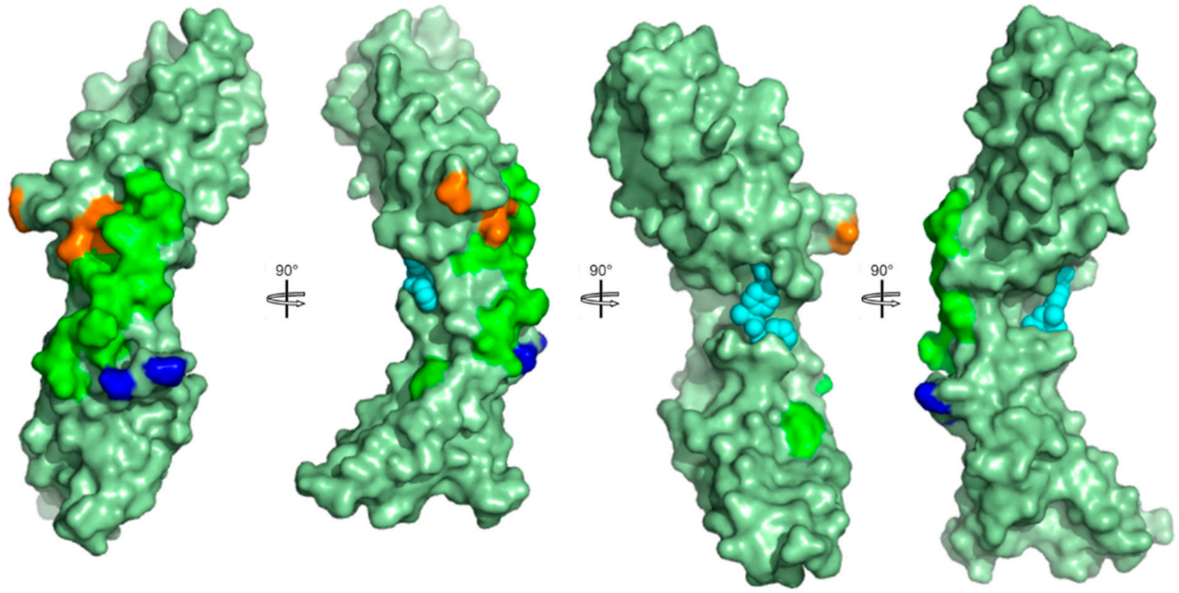

Figure S6. 3D "surface" representation of IZUMO1 and the famoxadone molecule (in light blue, spacefill). In orange, green and blue the surface residues involved in the binding to JUNO in the IZUMO 4HB domain, β-hairpin and Ig-like domain, respectively. The structures are rotated 90° to the right with respect to the X axis.

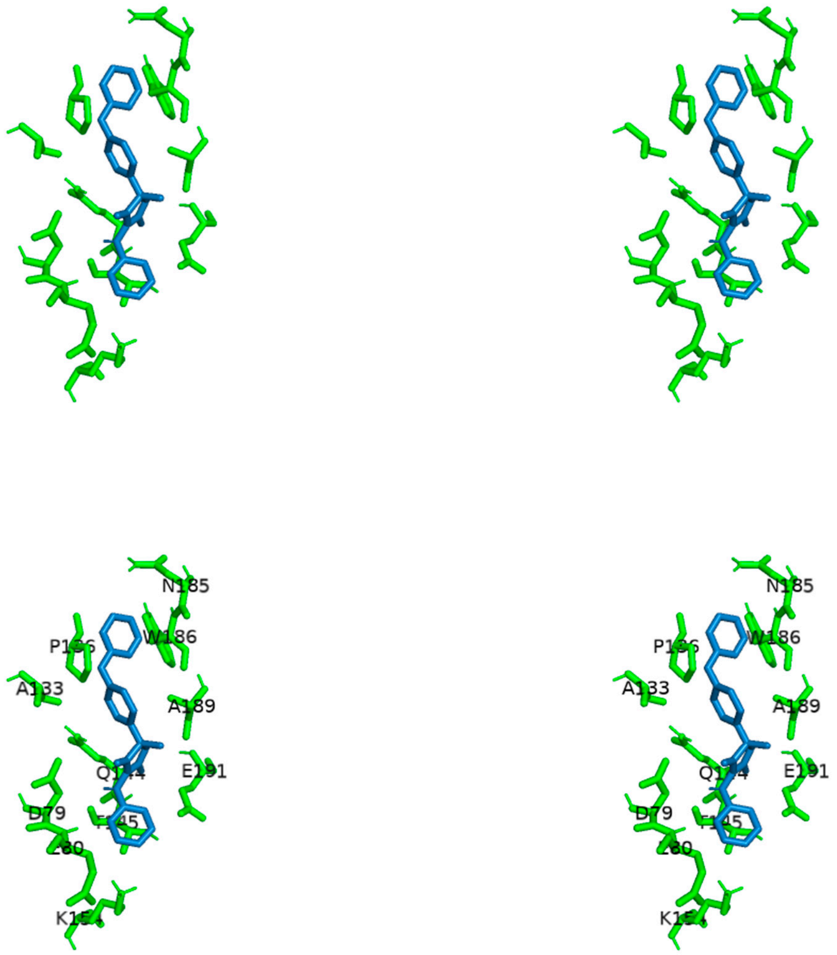

Figure S7. Stereo view in stick representation of IZUMO1 (PDB ID 5F4E) amino acid network (in green) surrounding the famoxadone structure (light blue) at a distance ≤ 4 Å.
